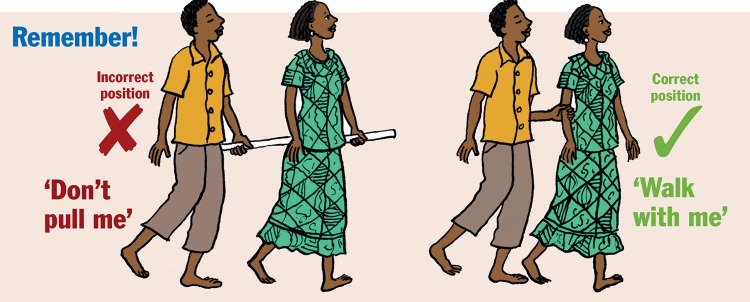# Assisting people who are visually impaired

**Published:** 2013

**Figure:**
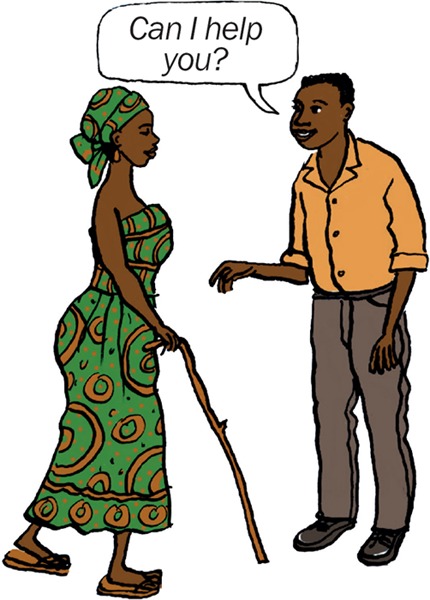
**Meeting and greeting** Always treat a person with impaired vision as you woudl anyone else. Introduce yourself first before offering help.

**Figure:**
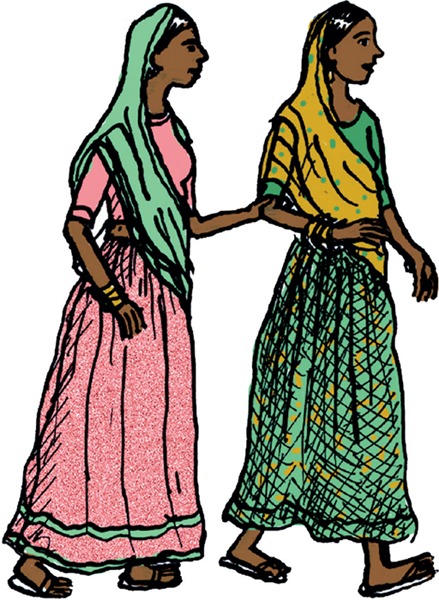
**Guiding** Walk side by side. Allow the person with impaired vision to set the pace and to hold your elbow (hand to elbow).

**Figure:**
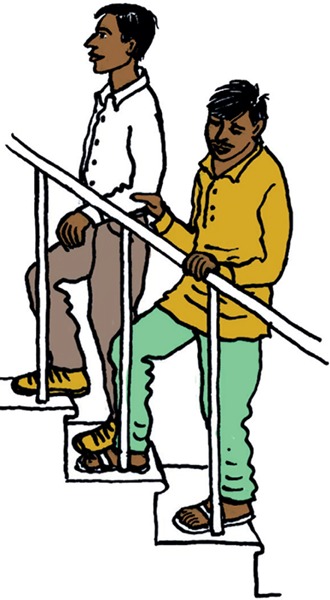
**Steps, stairs and slopes** Tell the blind or visually impaired person whether you are going up or down, and allow time for him/her to hold the handrail. Go one step ahead and take a slightly longer stride on the last step to allow your partner space.

**Figure:**
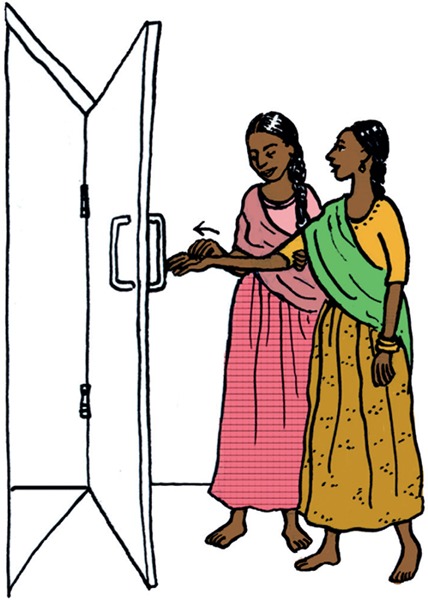
**Doorways** Tell the person whether the door opens towards or away from you. Go through the door with your partner on the side of the hinge. Open the door with your guiding hand. AI low your partner to feel the handle, follow you through the door, and close the door behind both of you.

**Figure:**
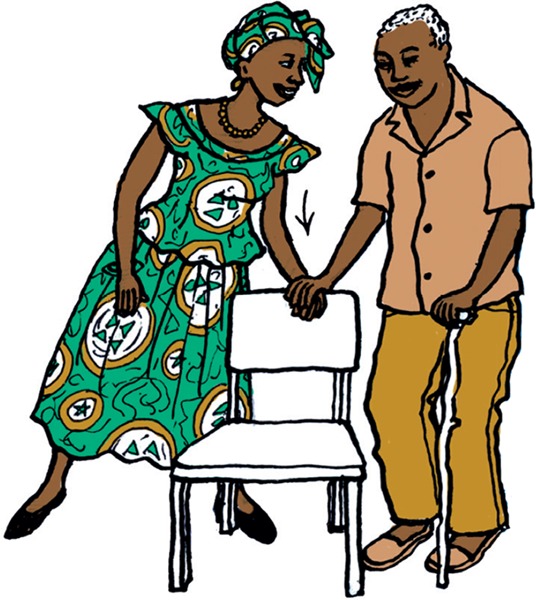
**Seating** Guide the person to the seat and explain what type it is (upright chair, low sofa, armchair, or stool). Ask him/her to let go of your arm and place a hand on the seat back or on the seat itself. He/she will now be able to judge its height and sit down safely.

**Figure:**
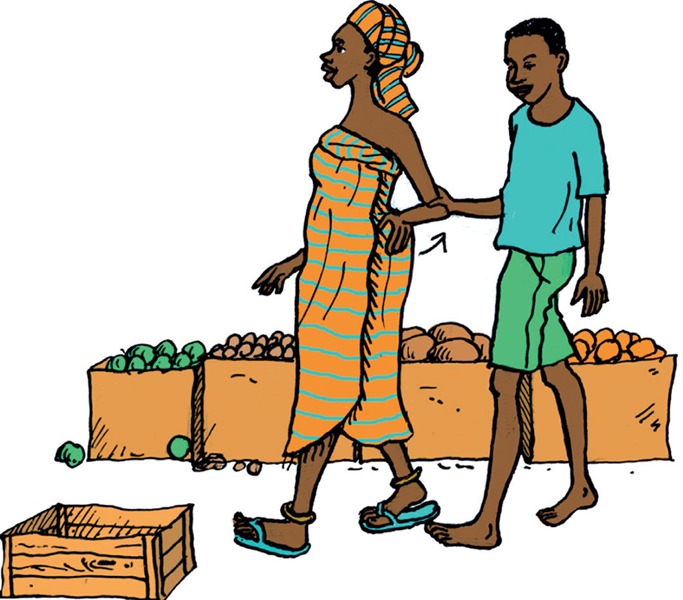
**Narrow spaces** Tell the person about the change in surroundings and then move your own guiding arm towards the middle of your back. Your partner should automatically step in behind you.

**Figure:**
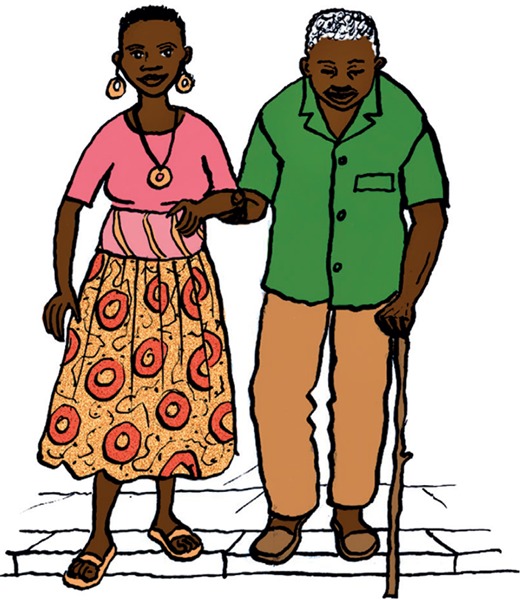
**Roads and kerbs** Tell the person if you are approaching ‘kerb up’ or ‘kerb down’ (the step onto or off a pavement or sidewalk), and pause slightly before taking the step. Cross the road using the shortest distance and go straight across.

**Figure:**
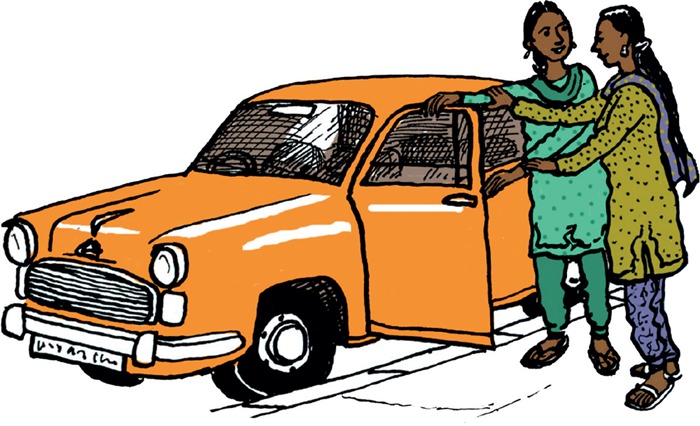
**Travelling by car** Tell the person if he/she is getting into the back or the front seat of the car, and whether it is facing left or right. Place your guiding hand on the door handle and allow him/her to slide his/her grip hand down your arm to the door handle. With the other hand he/she will be able to note the car roof and lower his/her head appropriately. At the end of the journey, get out first and help your partner out.

**Figure:**